# Intelligence and memory outcomes within 10 years of childhood convulsive status epilepticus

**DOI:** 10.1016/j.yebeh.2019.03.039

**Published:** 2019-06

**Authors:** Marina M. Martinos, Suresh Pujar, Helen O'Reilly, Michelle de Haan, Brian G.R. Neville, Rod C. Scott, Richard F.M. Chin

**Affiliations:** a Developmental Neurosciences Programme, UCL Institute of Child Health, London, UK; b Department of Neurological Sciences, University of Vermont, VT, USA; c Muir Maxwell Epilepsy Centre, University of Edinburgh, Edinburgh, UK

**Keywords:** PFS, prolonged febrile seizures, CSE, convulsive status epilepticus, NLSTEPSS, North London Convulsive Status Epilepticus in Childhood Surveillance Study, FSIQ, full scale intelligence quotient, GMS, Global memory scores, ICV, intracranial volume, Convulsive status epilepticus, Prolonged febrile seizures, Cognition, Memory, Long-term outcomes, Intracranial volume

## Abstract

Long-term intelligence and memory outcomes of children post convulsive status epilepticus (CSE) have not been systematically investigated despite evidence of short-term impairments in CSE. The present study aimed to describe intelligence and memory outcomes in children within 10 years of CSE and identify potential risk factors for adverse outcomes. In this cohort study, children originally identified by the population-based North London Convulsive Status Epilepticus in Childhood Surveillance Study (NLSTEPSS) were prospectively recruited between July 2009 and February 2013 and invited for neuropsychological assessments and magnetic resonance imaging (MRI) scans. Full-scale intelligence quotients (FSIQs) were measured using the Wechsler Abbreviated Scales of Intelligence (WASI), and global memory scores (GMS) was assessed using the Children's Memory Scale (CMS). The cohort was analyzed as a whole and stratified into a prolonged febrile seizures (PFS) and non-PFS group. Their performance was compared with population norms and controls. Regression models were fitted to identify predictors of outcomes. With a mean of 8.9 years post-CSE, 28.5% of eligible participants were unable to undertake testing because of their severe neurodevelopmental deficits. Children with CSE who undertook formal testing (N = 94) were shown to have significantly lower FSIQ (p = 0.001) and GMS (p = 0.025) from controls; the PFS group (N = 34) had lower FSIQs (p = 0.022) but similar memory quotients (p = 0.88) with controls. Intracranial volume (ICV), developmental delay at baseline, and active epilepsy at follow-up were predictive of long-term outcomes in the non-PFS group. The relationship between ICV and outcomes was absent in the PFS group despite its presence in the control and non-PFS groups. Post-CSE, survivors reveal significant intelligence and memory impairments, but prognosis differs by CSE type; memory scores are uncompromised in the PFS group despite evidence of their lower FSIQ whereas both are compromised in the non-PFS group. Correlations between brain volumes and outcomes differ in the PFS, non-PFS, and control groups and require further investigation.

## Highlights

• Aetiology is the overarching predictor of long term cognitive outcomes with intracranial volume, active epilepsy at follow-up, and developmental delay at baseline CSE being associated with worse outcomes ten years post CSE.
• Children who had PFS perform within the average range on standardized intelligence and memory assessments within ten years of CSE.
• Long term intelligence outcomes were associated with intracranial volumes in the non PFS and control groups but not the PFS group.

## 1 Introduction

Convulsive status epilepticus (CSE) is the most common neurological emergency in childhood [1] and is associated with a greater risk of pediatric mortality [2], structural brain abnormalities [3], [4], [5], [6], [7], [8], [9], [10], [11], [12], [13], [14], and an overall poorer quality of life [15]. It is becoming increasingly evident that children with CSE, even those with no apparent neurological problems prior to CSE, show evidence of short-term structural and functional complications after CSE [4], [5], [8], [9], [10], [11], [14]. Long-term outcomes after CSE in childhood are unclear; most studies that have been conducted to investigate this issue are either hospital-based, conducted retrospectively, and/or have involved both adults and children [16], [17], [18]. Only one prospective population-based study has focused on long-term outcomes of CSE in childhood, which, nevertheless, utilizes broad measures and does not investigate memory outcomes [19].

Our group carried out a 10-year follow-up study (the Status Epilepticus in Childhood Outcomes Study (STEPSOUT)) that was specifically designed to determine the prevalence, spectrum, clinical, and sociodemographic predictors of neurobehavioral outcomes of childhood CSE utilizing standardized assessments [20]. The inception cohort consisted of children identified and studied in the first-ever population-based study focused on the epidemiology of childhood CSE, i.e., the North London Convulsive Status Epilepticus in Childhood Surveillance Study (NLSTEPSS) [21].

From STEPSOUT, we have already reported on a higher risk of neurological problems, epilepsy, and behavioral issues in children who had CSE compared with the general population, with children who had prolonged febrile seizures (PFS) having a better prognosis than children whose initial episode of CSE was non-PFS [21], [22]. In the present paper, we report our findings on intelligence and memory outcomes as well as the factors associated with lower intelligence and memory abilities within 10 years of CSE. We present our results stratified according to All CSE, PFS, and non-PFS cases. Given our previous research where we studied children within a year of CSE [4], [5], [8], [9], we hypothesized that within 10 years post-CSE, (a) children with all forms of CSE would show deficits relative to controls and population norms and (b) children with non-PFS would be more affected than those who had PFS.

## 2 Methods

### 2.1 Participants and procedures

In this prospective cohort study, we targeted recruitment of all surviving children originally identified during the NLSTEPSS [21] (detailed recruitment methods described elsewhere) [20]. Children were classified into All CSE, and subsequently stratified according to etiology into PFS and non-PFS. Prolonged febrile seizure was defined as a seizure lasting longer than 30 min and occurring between the ages of 6 months and 5 years in the presence of fever and not caused by an acute or a remote insult to the central nervous system.

For the recruitment of healthy controls, we sent all-user emails to employees of Great Ormond Street Hospital (GOSH), a specialist tertiary care hospital based in London, and Young Epilepsy (YE), a national epilepsy charity. Parents volunteered participation of their children. Exclusion criteria for healthy control participation were a diagnosis of seizures/epilepsy and/or the presence of neurodevelopmental delay, as reported by their parents. We obtained written informed consent from all participants' parents/guardians, and where appropriate, we obtained assent from participants themselves. To examine the influence of socioeconomic status (SES) on outcomes, indices of multiple deprivation (IMDs) based on residential postal codes were determined for all participants (http://www.ons.gov.uk) as a measure of their SES.

### 2.2 Assessments

All participants were invited for a magnetic resonance imaging (MRI) and neuropsychological assessments at UCL Great Ormond Street Institute of Child Health (ICH) in London. The Wechsler Abbreviated Scales of Intelligence (WASI) [23] was used to obtain a full-scale intelligence quotient (FSIQ), a verbal intelligence quotient (VIQ), and a performance intelligence quotient (PIQ). The Children's Memory Scale (CMS) [24] was used to obtain a global memory scores (GMS). Both tests have a normative mean of 100 and a standard deviation of 15.

Brain MRIs were carried out on an Avanto 1.5 Tesla scanner (Siemens, Erlangen, Germany) using conventional brain MRI sequences. This was done for the following: (a) to assess the presence of structural brain abnormalities in this cohort and (b) to extract hippocampal and intracranial volume (ICV) measurements to investigate their relationship with memory and cognitive indices. Images were reviewed by an experienced pediatric neuroradiologist who determined whether scans were (1) normal, (2) contained a minor abnormality (abnormal feature thought to be either unrelated to this CSE episode/no functional significance), or (3) contained a major abnormality (abnormal feature likely to have significant impact on the child/represent a cause for this CSE episode) [25]. Two of the authors (SP and MM), blind to all clinical details, carried out the manual tracing of each hippocampus on 3D fast low angle shot (FLASH) images. Their measurements were averaged to arrive at a mean hippocampal value (HV). Intracranial volume was calculated using the brain extraction tool in FSL (https://fsl.fmrib.ox.ac.uk/fsl/fslwiki).

### 2.3 Statistical analysis

Analyses were conducted with Predictive Analytics Software (PASW) version 21 (Chicago, IL, U.S.A.) for Windows. Independent sample t-tests and chi-squared tests were used for intergroup comparisons. Univariate analysis of variance (ANOVA) controlling for age and SES was used to compare PFS, non-PFS, and control groups on FSIQ score and GMS; analysis was restricted to FSIQ rather than its constituents VIQ and PIQ as we did not observe any discrepancies between VIQ and PIQ in our sample. One sample t-tests were conducted to compare CSE group means with the population norms. Significant difference in test results was set at p < 0.05.

To identify factors associated with lower intelligence and memory scores according to etiological classification, we performed separate regression analyses for all patients with CSE, PFS, and non-PFS. To assess whether any CSE features were directly related to outcome, we investigated the following in the regression analyses: (a) duration of CSE (min), (b) CSE type (intermittent versus continuous), and (c) CSE loci (focal versus generalized). In addition, given past literature in this field, we also investigated whether (d) age at CSE, (e) recurrence of CSE, (f) active epilepsy at follow-up, (g) cognitive delay at baseline (yes/no), (h) motor delay at baseline (yes/no), (i) previous seizures at baseline (febrile, afebrile/epilepsy, no seizures), (j) MRI visible structural abnormalities at follow-up (no/minor abnormalities/major abnormalities), and (k) prematurity (yes/no) affected outcomes. Antiepileptic drugs were not entered as an independent variable as they are highly correlated with active epilepsy at follow-up. Finally, given the well-established role of the hippocampus in cognition, we also included (l) mean HV (mm^3^) in our regression analyses as well as (m) ICV (mm^3^), which served as a proxy for total brain volume. Bootstrapping of 1000 samples was applied to all our analyses. Any factors significant on univariable analyses at the p < 0.1 level were subsequently included in multivariable modeling (stepwise regression) to identify the factors associated with neurocognitive outcomes after adjustment of others.

Finally, given the nature of the study population, we anticipated that some participants would be unable to participate in our neuropsychological assessment because of their severe developmental delay. Such data would not have been missing at random. The overhauling majority of participants who were not assessable on the WASI and the CMS had non-PFS (bar one child with PFS). To address this matter, we imputed the minimum value (FSIQ = 50) for each participant with non-PFS that was not assessable on the WASI; a similar strategy has been used in other studies of children with seizures [26]. Imputed FSIQ scores were only applied in the regression analysis to compare with findings from the completed-case approach.

## 3 Results

### 3.1 Participant characteristics

**Fig. 1:**
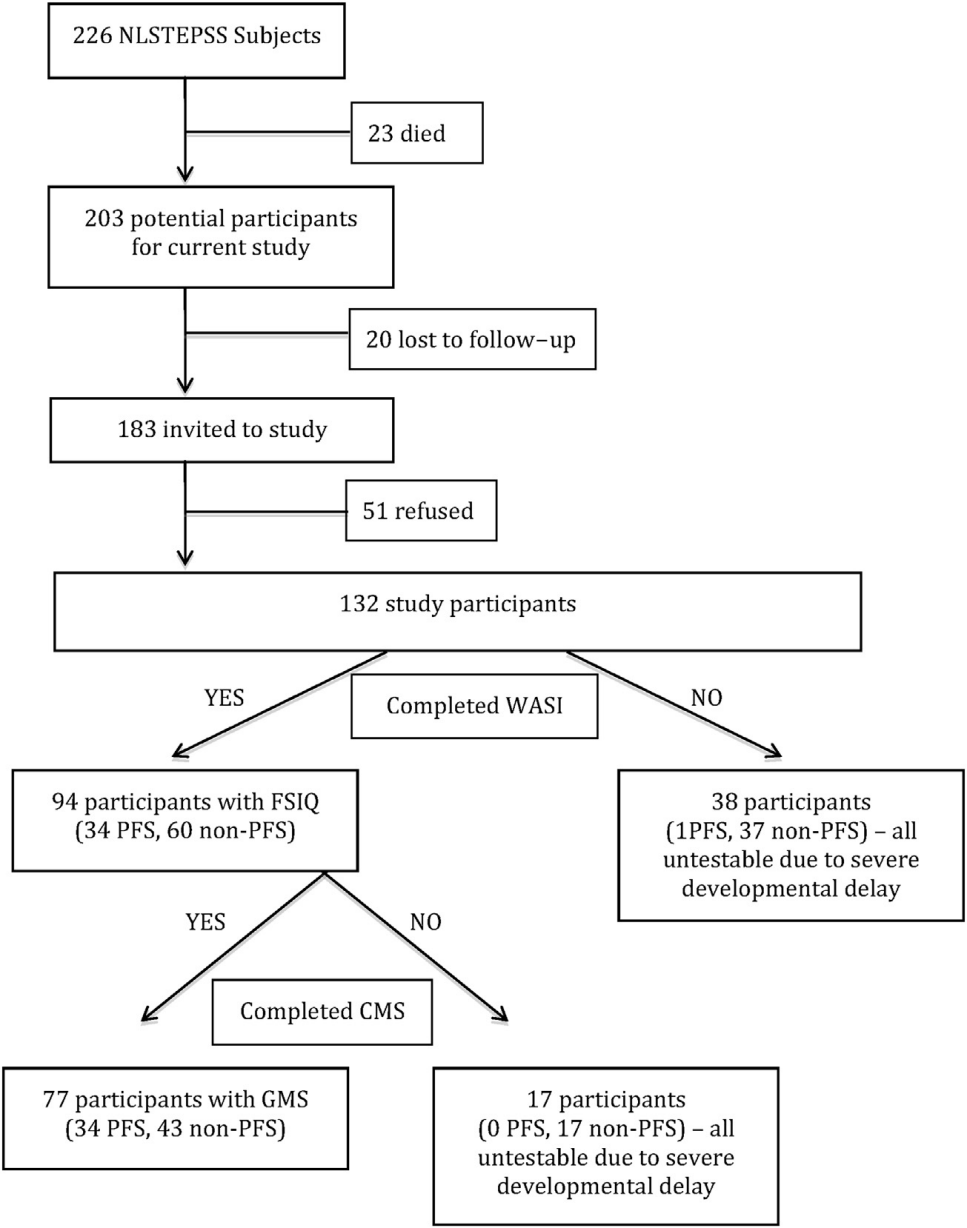
Flow chart of recruitment and assessments. WASI = Wechsler Abbreviated Scales of Intelligence, FSIQ = full-scale intelligence quotient, CMS = Children's Memory Scale, GMS = global memory score, PFS = prolonged febrile seizure, and non-PFS = nonprolonged febrile seizure.

We identified 183 (90%) of the 203 survivors from the inception CSE cohort for participation in the current study; 20 subjects were lost to follow-up. One hundred and thirty-two children (65% of inception cohort survivors) participated in the study (i.e., agreed to take part in neuropsychological assessments and provided clinical data), forty-nine refused study participation altogether, and two provided follow-up data but refused neuropsychological and MRI scanning (51 refusals in total) (see Fig. 1 for flow chart of study recruitment). From the 132 participants, a bigger proportion was preterm and had generalized-onset CSE compared with the 71 nonparticipants (51 refusals and 20 lost to follow-up). The two groups were similar in all other CSE-related, clinical and demographic variables (Supplemental Table 1).

From the 132 participants, 94 children (34 PFS, 60 non-PFS) were assessed with the WASI, a mean of 8.6 years following CSE to derive a FSIQ. From the remaining participants, 38 (1 PFS, 37 non-PFS) were not assessed with our neuropsychological instruments, due to their severe developmental delay (2 out of these 38 children had an MRI). Children with PFS who undertook neuropsychological testing (N = 34) were similar to the nonparticipating PFS survivors (N = 22) for all variables except for a larger representation of females in study participants (χ^2^ (1) = 5.04, p = 0.03). In the non-PFS group of children who completed the FSIQ assessment (N = 60), there was a significantly smaller proportion of children with cognitive delay (χ^2^ (1) = 15.31, p < 0.001) and motor delay at baseline (χ^2^ (1) = 7.57, p = 0.007) than children who did not participate (N = 87). Moreover, children with non-PFS classified as having any type of seizures (i.e., febrile or afebrile) prior to CSE baseline were less likely to participate in our neuropsychological assessments (χ^2^ (1) = 12.4, p = 0.002).

Seventy-seven children (34 PFS, 43 non-PFS) out of 94 children who were assessed on the WASI were also tested on the CMS. Seventeen non-PFS cases could not be assessed on the CMS because of their severe learning difficulties (mean FSIQ difference between participants able to complete CMS (mean: 94.6) and those unable (mean: 60.2): t (92) = − 9.4, p < 0.001).

Seventeen neurologically healthy control subjects without a history of febrile or afebrile seizures (see Table 1 for group characteristics) provided CMS and WASI data for comparison.

**Table 1:** Demographic and clinical characteristics of study participants who were able to complete a FSIQ assessment (n = 94) compared with all (n = 109) other survivors of the inception cohort who did not have FSIQ assessment (n = 109: 20 lost to follow-up, 51 study refusals, and 38 untestable), and controls (n = 17).

	Study participants who completed FSIQ assessment (N = 94)		Survivors of inception cohort without FSIQ assessment (N = 109)		Controls (N = 17)
	PFS (N = 34)	Non-PFS (N = 60)	PFS (N = 22)	Non-PFS (N = 87)	(N = 17)
Gender (female:male)	18:16	27:33	5:17	51:36	10:7
Age at CSE in months (SD)	19.38 (14.08)	50.36 (37.35)	23.41 (12.07)	62.46 (50.55)	–
Age at follow-up in years (SD)	10.04 (1.51)⁎	12.89 (3.39)⁎	–	–	12.37 (3.10)⁎
IMD at CSE (SD)	33.52 (16.99)	32.72 (14.40)	31.88 (12.56)	35.3 (13.66)	24.16 (17.71)
Full-term (> 36 weeks) (%)	30 (88.2%)	48 (80%)	19/20 (95%)	66/77 (85.7%)	54/59 (78.3%)
Diagnosis of epilepsy (before or during follow-up) (%)	5 (14.7%)	39 (65%)	–	–	–
Seizures prior to CSE (%)	11 (32.4%)	33 (55.5%)	8 (36.4%)	64/86 (74.4%)	–
Motor delays at CSE baseline (%)	0	12 (20%)	0	34/81 (42%)	–
Cognitive delays at CSE baseline (%)	0	29/59 (49.2%)	0	52/73 (71.2%)	–
CSE duration in minutes (SD)	83.5 (89.26)	88.7 (73.75)	80.91 (50.06)	92.79 (111.70)	–
Focal CSE (%)	6 (17.6%)	28 (46.7%)	7 (31.8%)	38 (43.7%)	–
Continuous CSE (%)	18 (52.9%)	29(48.3%)	12 (54.5%)	40 (46%)	–
CSE recurrence (%)	10 (29.4%)	27 (45%)		–	–
Normal MRI at follow-up (%)	29 (85.3%)	28/54 (46.7%)	–	–	17 (100%)
Mean HV measured at follow-up (SD)	3210.35 mm^3^ (361.6)⁎	2949.54 mm^3^ (425.8)⁎	–	–	3124.96 mm^3^ (248.12)⁎
ICV measured at follow-up (SD)	1,470,954.6 mm^3^ (150,687.5)	1,453,130.74 mm^3^ (228,040.9)	–	–	1,479,983.82 mm^3^ (121,489.440)

Abbreviations: prolonged febrile seizures (PFS), convulsive status epilepticus (CSE), index of multiple deprivation (IMD), intracranial volume (ICV), hippocampal volume (HV), magnetic resonance imaging (MRI), standard deviation (SD), and North London Convulsive Status Epilepticus in Childhood Surveillance Study (NLSTEPSS).

⁎ Significant results at the p < 0.05 level after Bonferroni correction for ANOVA comparisons between the PFS participants, non-PFS participants, and controls.

### 3.2 Neuropsychological results

#### 3.2.1 All CSE group comparisons

With a mean of 8.6 years post-CSE, children had significantly lower FSIQ (t = 4.24, p = 0.001; d = 1.23) and GMS (t = 1.92, p = 0.025; d = 0.54) scores than controls (see Table 2).

**Table 2:** Results from the WASI and the CMS for all groups.

	All CSE	PFS	Non-PFS	Controls
FSIQ				
N	94	34	60	17
Mean	88.4	100.4	81.6	111.5
Standard deviation	21.4	13.8	22	15.8
Range	50–134	74–120	50–134	82–138

VIQ				
N	93	34	59	17
Mean	89.03	100.1	82.7	110.5
Standard deviation	20.73	12.04	22.04	15.99
Range	55–131	74–123	55–131	74–129

PIQ				
N	92	34	58	17
Mean	89.7	100.8	83.2	109.88
Standard deviation	20.6	15.96	20.3	15.26
Range	53–134	68–131	53–134	88–145

GMS				
N	77	34	43	17
Mean	96.4	105.7	89	107.4
Standard deviation	22.2	17.5	22.9	18
Range	50–134	67–133	50–134	68–137

Abbreviations: Wechsler Abbreviated Scales of Intelligence (WASI), full-scale intelligence quotient (FSIQ), Children's Memory Scale (CMS), global memory scores (GMS), prolonged febrile seizures (PFS), convulsive status epilepticus (CSE), and number of participants (N).

#### 3.2.2 Group comparisons stratified by PFS and non-PFS

Mean FSIQ was lowest in children who had non-PFS, higher in children who had PFS, and highest in controls (see Table 2). Univariate ANOVA comparing the PFS, the non-PFS, and the control groups on FSIQ scores with age and SES as covariates revealed an effect of group (F = 17.13, p < 0.001, η^2^ = 0.24; power = 1). Post-hoc comparisons with Bonferroni corrections revealed that the PFS group had lower FSIQ scores compared with controls (p = 0.022) but higher FSIQ scores compared with the non-PFS group (p = 0.001). In turn, the non-PFS group, had lower FSIQ scores than the control group (p = 0.001).

The mean GMS was also lowest in the non-PFS group, higher in the PFS group, and highest in controls (Table 2) with univariate ANOVA with age and SES as covariates confirming an effect of group (F = 6.75, p = 0.002, η^2^ = 0.13; power = 0.91). The PFS group's mean GMS was not significantly different to controls (p = 0.9), but GMS were higher in the PFS group compared with the non-PFS group (p = 0.002) while the non-PFS group had lower scores than controls (p = 0.002).

#### 3.2.3 Comparison with test derived means

The All CSE group obtained lower scores than the normative mean for FSIQ (t = − 5.25, p = 0.001; d = − 0.54, power: 0.99), VIQ (t = − 5.10, p < 0.001; d = − 0.53), and PIQ (t = − 4.8, p < 0.001; d = − 0.50) but did not differ from the test-provided GMS means (t = − 1.44, p = 0.15; d = − 0.16). The PFS group obtained FSIQ (d = 0.03), VIQ (d = 0.008), and PIQ (d = 0.05) scores that were not significantly different from the normative mean (p > 0.05), nor was there any difference in GMS with the normative mean (t = 1.9, p = 0.07; d = 0.33, power: 0.48). The non-PFS group performed significantly worse than the normative mean for FSIQ (t = − 6.48, p < 0.001; d = − 0.84, power: 1), PIQ (t = − 6.17, p < 0.001; d = − 0.78, power: 0.1), and VIQ (t = − 5.73, p < 0.001; d = − 0.83, power: 0.1) as well as the GMS (t = − 3.15, p = 0.003; d = − 0.48, power: 0.9).

### 3.3 Regression results

The results of the univariable regression analyses can be found in Table 3 and Supplemental Table 2. In the All CSE group analysis, lower FSIQ scores were independently associated with smaller ICVs at follow-up (B = 2.78, p = 0.008), cognitive delay at baseline CSE (B = − 13.31, p = 0.02), the occurrence of previous seizures at CSE (B = − 5.16, p = 0.05), and the presence of active epilepsy at follow-up (B = − 11.72, p = 0.06) (R^2^ = 0.51, p < 0.001). Lower GMS were associated with the occurrence of previous seizures at the time of CSE (B = − 9.21, p = 0.007) and cognitive delay at baseline CSE (B = − 23.66, p = 0.001) (R^2^ = 0.36, p < 0.001).

**Table 3:** Results from the univariable regression for the three participant groups.

	PFS	Non-PFS	Controls
*FSIQ*			
Age at CSE in months	B = − 0.13, p = 0.46	B = − 0.06, p = 0.46	N/A
Prematurity (< 36 weeks)	B = − 2.48, p = 0.74	B = − 14.5, p = 0.01⁎	N/A
MRI visible abnormalities	B = − 9.06, p = 0.22	B = − 12.2, p = 0.04⁎	N/A
Seizures prior to CSE	B = − 9.52, p = 0.07&	B = − 14.25, p = 0.001⁎	N/A
Active epilepsy	N/A	B = − 26.467, p < 0.001⁎	N/A
Motor delays at CSE	N/A	B = − 29.58, p < 0.001⁎	N/A
Cognitive delays at CSE	N/A	B = − 33, p < 0.001⁎	N/A
Duration of CSE	B = − 0.02, p = 0.41	B = − 0.58, p = 0.06&	N/A
Focal CSE	B = 1.29, p = 0.81	B = − 5.41, p = 0.38	N/A
Continuous CSE	B = 4.13, p = 0.37	B = − 3.98, p = 0.5	N/A
CSE recurrence	B = − 4.59, p = 0.41	B = − 17.99, p = 0.001⁎	N/A
Mean HV at follow-up	B = − 0.01, p = 0.13	B = 0.02, p = 0.001⁎	B = 0.01, p = 0.5
ICV	B = 9.24, p = 0.64	B = 5.68, p = 0.003⁎	B = 8.8, p = 0.002⁎

*GMS*			
Age at CSE in months	B = − 0.023, p = 0.93	B = − 0.01, p = 0.92	N/A
Prematurity (< 36 weeks)	B = − 5.58, p = 0.59	B = − 16.89, p = 0.05⁎	N/A
MRI visible abnormalities	B = − 15.83, p = 0.03⁎	B = − 8.13, p = 0.32	N/A
Seizures prior to CSE	B = − 12.15, p = 0.08&	B = − 11.85, p = 0.005⁎	N/A
Active epilepsy	N/A	B = − 20.988, p = 0.065&	N/A
Motor delays at CSE	N/A	B = − 28.47, p = 0.003⁎	N/A
Cognitive delays at CSE	N/A	B = − 30.32, p < 0.001⁎	N/A
Duration of CSE	B = − 0.03, p = 0.42	B = − 0.09, p = 0.22	N/A
Focal CSE	B = 6.06, p = 0.33	B = − 14.61, p = 0.042⁎	N/A
Continuous CSE	B = 4.86, p = 0.43	B = − 13.74, p = 0.06&	N/A
CSE recurrence	B = − 11.73, p = 0.12	B = − 17.97, p = 0.013⁎	N/A
Mean HV at follow-up	B = − 0.01, p = 0.11	B = 0.02, P = 0.004⁎	B = − 0.02, p = 0.42
ICV at follow-up	B = − 3.10, p = 0.89	B = 4.62, p = 0.066&	B = 4.89, p = 0.1

Abbreviations: Prolonged febrile seizures (PFS), convulsive status epilepticus (CSE), socioeconomic status (SES), intracranial volume (ICV), hippocampal volume (HV), magnetic resonance imaging (MRI), and Not applicable (N/A).

⁎ p < 0.05.

& p < 0.10.

For the PFS group, no variable was retained in the multivariable model. For the non-PFS group, a smaller ICV at follow-up (B = 4.02, p = 0.002), active epilepsy at follow-up (B = − 16.96, p = 0.005), and cognitive delay at CSE baseline (B = − 3.22, p = 0.011) were associated with lower FSIQ scores (R^2^ = 0.57, p < 0.001). The same result was obtained in our all-inclusive non-PFS regression analysis (R^2^ = 0.61, p < 0.001). Lower GMS were associated with cognitive delay at baseline (B = − 29.14, p < 0.001) (R^2^ = 0.33, p < 0.001).

For the control group, at the multivariable level, ICV was predictive of FSIQ (R^2^ = 0.46, p = 0.003).

## 4 Discussion

This novel prospective study investigated intelligence and memory outcomes in a population-based cohort within ten years post-CSE. Children with a history of CSE were shown to have lower FSIQ scores and GMS than controls as well as population norms. This difference was primarily driven by children classified as non-PFS; those classified as PFS had lower FSIQs than controls but scored in the average range on the WASI. Their GMS were on a par with controls and population norms.

Sixty-five percent of children classified as non-PFS received a diagnosis of epilepsy at the time of their initial CSE or during their follow-up; this clinical factor proved to be significantly associated with their intelligence outcomes. In a recent study, 40% of children with active epilepsy were shown to obtain FSIQs below 70 pointing to the intellectual vulnerability of this group [27]. In the present study, we also report a recurring association [5] between the presence of previous seizures at CSE and a worse long-term outlook in this population. This result may be explained by the “multiple insult” hypothesis whereby CSE plus more seizures equals worse outcomes than CSE alone or seizures alone [28]. Alternatively, it can be argued that the occurrence of previous seizures at CSE indicates a graver underlying pathology and could serve as a useful biomarker for clinicians for identifying the most “at-risk of long-term sequelae” cases.

A better long-term neurocognitive outcome in children with non-PFS was predicted by larger brain volumes, marked in the present study by ICV measured at follow-up, as well as the absence of cognitive delay at baseline. The relationship between ICV and intelligence is well documented in the literature [29], [30] and is perhaps not surprising in the current context. Namely, smaller brain volumes at baseline CSE could be a marker for worse concomitant neurological problems. Conversely, a larger brain might be associated with increased plasticity following an initial insult. This is supported by a recent study in guppies that reports that a bigger brain size leads to increased levels of neuroplasticity [31]. The finding that cognitive delay at baseline predicts neurocognitive outcomes within ten years is consistent with our earlier findings where (a) signs of developmental problems prior to CSE were predictive of outcomes within 6 weeks and (b) cognitive composite and language scores obtained at 6 weeks were strongly correlated to overall outcome at one year [9].

The PFS group obtained significantly higher FSIQs than the non-PFS group and significantly lower FSIQs than healthy controls mirroring ours and others' findings within one year of CSE [9]. The finding that FSIQs were similar to population norms in this group may be a result of the Flynn effect; a tendency of IQ scores to increase over time and the failure of measuring tools to keep up with this change [32]. Another potential contributing consideration is that the WASI, which we used to measure FSIQ in the present study, tends to overestimate FSIQ compared with the Wechsler Intelligence Scale for Children IV [33]. Thus, the current findings may reflect an overestimation of this group's abilities in their intellectual functions. However, the present findings serve to corroborate previous reports of the absence of intellectual disabilities in this group. Whether learning difficulties are more prevalent in this population would require more in depth investigations using instruments such as the Wechsler Individual Achievement test [34].

Since we have previously shown that incidental recognition memory was reduced within 6 weeks as well as one year post-PFS [8], it was unexpected to find that the PFS group was no different to controls or population norms on the CMS in the current study. A potential explanation for this discrepancy could be that the incidental recognition memory paradigm adopted for our early outcomes study recruits different brain structures and mental processes than the CMS. This has been indicated in several past studies that have demonstrated dissociations in performance on incidental recognition and explicit recognition tasks [35], [36], [37]. In support of this, our original one-year outcome data showed no significant correlations between CMS and incidental recognition memory scores in the 8 individuals where both measures were collected [38]. Moreover, findings obtained from our early outcome studies might have picked up on transient cognitive changes resulting from the CSE incident. To adjudicate between these two possibilities, we would need to administer an incidental recognition paradigm to our long-term follow-up CSE cohort.

**Fig. 2:**
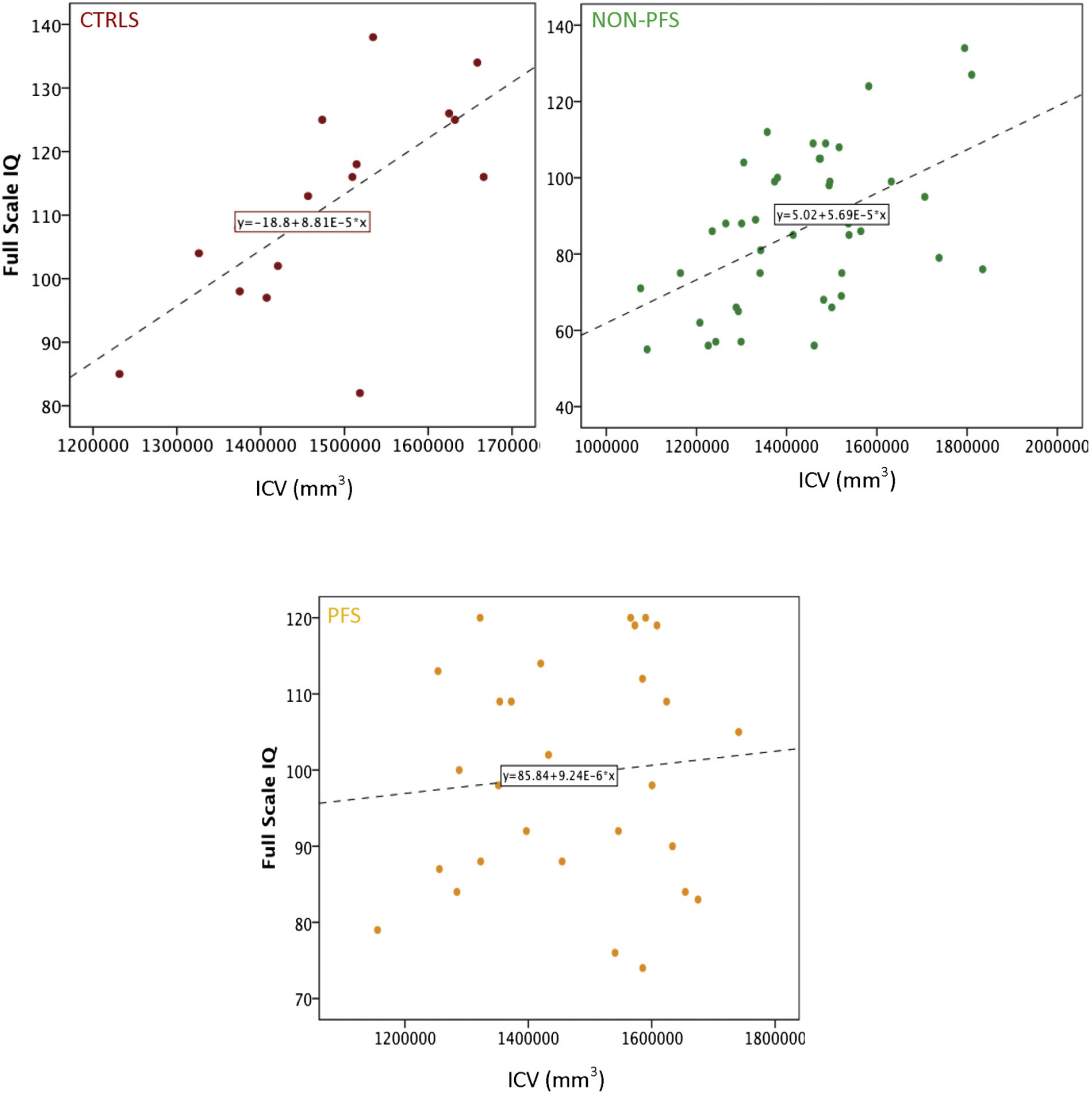
Relationship between ICV and FSIQ in the PFS, non-PFS, and control groups. Abbreviations: prolonged febrile seizures (PFS), controls (CTRLS), full-scale intelligence quotient (FSIQ), and intracranial volume (ICV).

Lastly, the absence of a relationship between ICV and outcomes in the PFS group despite its evidence in the non-PFS and control groups (see Fig. 2) was unexpected and requires further investigation. A possible explanation for this is that post-CSE, there is reorganization of cortical matter as well as microstructural reorganization [3], [38] that breaks down the typical brain function relationships in this group.

### 4.1 Study limitations

One possible limitation of the current study is attrition, with 34% of the inception cohort not providing follow-up data. Nevertheless, loss to follow-up is inevitable in long-term cohort studies, and the 34% dropout observed in the present study is considered acceptable for such long-term follow-up studies [39], [40], [41]. A comprehensive comparison of participants and nonparticipants on demographics and clinical characteristics revealed differences between the two groups solely in the proportion of children born prematurely and children who had a generalized-onset CSE versus a focal onset. The two groups were similar in all other variables suggesting that the studied group is a good representation of the entire cohort.

Secondly, 28.5% of our sample was unable to provide numerical data because of their inability to complete assessments. Nevertheless, rather than being a limitation, this attrition reflects the outlook of children following CSE associated with neurological complications and speaks to their pervasive difficulties. In addition, this level of attrition did not take place within the PFS group who, in their overhauling majority, undertook neuropsychological assessments (bar one child with intellectual disability and autism). Moreover, when we included the whole non-PFS group by substituting nonscores with the lowest possible FSIQ score, regression results remained unchanged suggesting that the predictors identified in our study are generalizable to the entire studied population.

Thirdly, it could be argued that we recruited a modestly sized control group that were the offspring of hospital and university employees and, consequently, may have been an intrinsically higher functioning group. Nevertheless, the CSE group as a whole was not statistically different in SES, gender, and age to our group of controls. In addition, we controlled for age and SES in all our subgroup analyses. On top of that, our overall findings remained consistent when comparisons were made with the instruments' population normative data arguing against this notion. Using the more recent edition of the WASI (i.e., WASI-II) may have been preferable as the older version (WASI-I), which was published in 1999, may have overestimated FSIQs in the present study. Nevertheless, study recruitment was well underway (July 2009–February 2013) when the WASI-II was released in 2011 [41].

Finally, with recent advances in the identification of certain genetic mutations (e.g., SCN1A), the current classification system may be obscuring further divisions within each group driven by their genetic heterogeneity. Thus, our findings should be considered keeping these limitations in mind.

## 5 Conclusion

Children within ten years of CSE reveal intelligence and memory impairments relative to controls and population norms, albeit these are strongly associated with the etiology of CSE. Children classified as non-PFS at baseline have a worse outcome associated with the presence of cognitive delay pre-CSE and ICV at follow-up. Children with a history of PFS reveal no memory impairments on the CMS.

## Appendix A Supplementary data

Supplementary tables Image 1
