# Supplementary material for: Intelligence and memory outcomes within 10 years of childhood convulsive status epilepticus
Source: Epilepsy Behav. 2019 Jun;95:18–25. doi: 10.1016/j.yebeh.2019.03.039 (PMC6586081; doi:10.1016/j.yebeh.2019.03.039)
Supplement: Supplementary file 1 — Supplementary tables [file mmc1.docx]

SUPPLEMENTARY MATERIAL

**Supplemental Table 1: Demographic and clinical characteristics of Study Participants and Dropouts (lost to follow up & study refusals)**

|  | **Study Participants (N=132)** | **Dropouts**  **(N=71)** | **Difference in proportion (95%CI)/Difference in Means or medians** |
| --- | --- | --- | --- |
| **Gender (Female: Male)** | 66:66 | 35:36 | 0.7 (-13.4, 14.8) |
| **Age at CSE in Months (SD)** | 46·2 (41) | 49·7 (46·5) | p=0.9 |
| **SES (SD)** | 33·3 (15) | 34·9 (12·9) | p=0.7 |
| **Ethnic Category** | 53 white: 79 non-white  22 black  40 Asian  7 mixed  10 other | 21 white: 50 non-white  18 black  22 Asian  4 mixed  6 other | 10.5 (-3.4, 23.2) |
| **Full term (>36 weeks)** | **107/130(82·3%)** | **56/61(91·8%)** | **-10.7 (-13.9, -7.3)** |
| **Seizures prior to CSE** | 76 (57·6%) | 41 (57·7%) | -0.2 (-13.9, 13.9) |
| **Normal motor development prior to CSE** | 83 (62·9%) | 45/64 (70·3%) | -7.4 (-20.4, 6.9) |
| **Normal cognitive development prior to CSE** | 68 (51·5%) | 37/64 (57·8%) | -6.3 (-18.3, 6.2) |
| **Duration of CSE in minutes (SD)** | 94·6 (110·2) | 77·8 (39·4) | p=0.2 |
| **Focal CSE (%)** | **46 (34·8%)** | **35 (49·3%)** | **-14.4 (-23.3, -5.2)** |
| **Continuous CSE (%)** | 68 (51·5%) | 31 (43·7%) | 7.8 (-6.5, 21.6) |

**Abbreviations:** Convulsive status epilepticus (CSE), socioeconomic status (SES), number (N), standard deviation (SD), confidence interval (CI).

**Supplemental Table 2 Results from the univariable regression for all CSE patients combined**

|  | **FSIQ** | **GMS** |
| --- | --- | --- |
| **Age at CSE in Months** | B=-0.17, p=0·009***** | B=-0.13, p=0·106 |
| **Prematurity (<36 weeks)** | B=-13.7, p=0·02***** | B=-15.6, p=0·054***** |
| **MRI visible abnormalities at follow-up** | B=-9.33, p=0·001***** | B=-10.40, p=0·004***** |
| **Seizures prior to CSE** | B=-15.62, p=0·001***** | B=13.34, p<0.001***** |
| **Active epilepsy at follow-up** | B=-31.39, p<0.001***** | B=-27.31, p=0.008***** |
| **Motor delays at CSE** | B=-34.05, p<0.001***** | B=-34.56, p<0.001***** |
| **Cognitive delays at CSE** | B=-7.70, p<0.001***** | B=-34.31, p<0.001***** |
| **Duration of CSE** | B=-0.43, p=0·13 | B=-0.03, p=0.438 |
| **Focal CSE** | B=-8.89, p=0·05***** | B=-10.69, p=0.062^&^ |
| **Continuous CSE** | B=-1.85, p=0·68 | B=-5.79, p=0·294 |
| **CSE recurrence** | B=-16.20, p<0.001***** | B=-17.567, p=0.003***** |
| **Mean HV at follow-up** | B=0.016, p=0.002***** | B=0.014, p=0.034***** |
| **Mean HV at follow up corrected for ICV** | B=3391.76, p=0.678 | B=7308, p=0.452 |
| **ICV at follow-up** | B=4.53, p<0.001***** | B=3.12, p=0.054***** |

***** p<0·05

^&^ p<0.10

**Abbreviations**: Full scale intelligence quotient (FSIQ), global memory score (GMS), convulsive status epilepticus(CSE), intracranial volume (ICV), hippocampal volume (HV), magnetic resonance imaging (MRI).
